# Supplementary material for: A novel rolling bearing fault detect method based on feature mode decomposition and subtraction-average-based optimizer
Source: PLoS One. 2025 Jun 4;20(6):e0324739. doi: 10.1371/journal.pone.0324739 (PMC12136367; doi:10.1371/journal.pone.0324739)
Supplement: Attachment — Submitted filename: Comments for PONE-D-24-49078R1.pdf [file pone.0324739.s004.pdf]

Comments to Editor

Authors has essentially addressed the issues raised previously, but there are still some shortcomings. Thus, I recommend a **major revision**. Here are my reasons and suggestions.

Comments to Authors

Authors has essentially addressed the issues raised previously, but there are still some shortcomings. Here are my suggestions that may help the authors to improve this study.

a) Why can the combination of SABO and FMD effectively perform feature extraction? Their respective characteristics need to be emphasized in the text.
b) The innovation of the article lies in combining SABO with FMD for feature extraction. How are they combined? Where exactly does the innovation manifest? Were there any difficulties encountered during their integration? Please emphasize these points in the text.
c) The first occurrence of an abbreviation should be accompanied by its full name, like SABO and FMD in the abstract; it is recommended that the citation number should not appear at the beginning of a sentence; the table layout is unreasonable, and the table is too large.
d) The abstract does not reflect the innovative aspects and the workflow of the paper.
e) Does the comparative method have any relevant references? If so, it is recommended to indicate them.
f) It is suggested to attach the figures behind the relevant text; otherwise, the readability will be very poor.
g) The descriptions of the images related to the experimental results are too brief. It is recommended to provide additional information.
h) It is recommended to increase the proportion of references from the most recent four years.
