# Supplementary material for: A novel rolling bearing fault detect method based on feature mode decomposition and subtraction-average-based optimizer
Source: PLoS One. 2025 Jun 4;20(6):e0324739. doi: 10.1371/journal.pone.0324739 (PMC12136367; doi:10.1371/journal.pone.0324739)
Supplement: Attachment — Submitted filename: Comment_PONE-D-24-49078.docx [file pone.0324739.s002.docx]

**Comments to Editor**

In this paper, a fault diagnosis method that integrates Subtraction Average-Based Optimizer (SABO) with FMD has been proposed. The approach employs the index of minimum envelope entropy as a fitness function to derive an optimal combination of FMD parameters through the SABO optimization algorithm, subsequently facilitating fault diagnosis via envelope spectrum analysis. Experiments show the effectiveness. The paper's content structure is complete, but there are issues with non-standard formatting and poor readability. Thus, I recommend a minor revision. Here are my reasons and suggestions.

**Comments to Authors**

In this paper, a fault diagnosis method that integrates Subtraction Average-Based Optimizer (SABO) with FMD has been proposed. The approach employs the index of minimum envelope entropy as a fitness function to derive an optimal combination of FMD parameters through the SABO optimization algorithm, subsequently facilitating fault diagnosis via envelope spectrum analysis. Experiments show the effectiveness. The paper's content structure is complete, but there are issues with non-standard formatting and poor readability. Here are my suggestions that may help the authors to improve this study.

a) The paper's format is not standardized enough, for example, there are deformations in the symbols and letters, the font in the figures is too small, and there are multiple blanks in the paper.
b) There is a lack of an overall scheme diagram, which reduces the readers' understanding of the methodological process of the paper.
c) The paper's methodology focuses on achieving accurate classification of rolling bearing faults from the perspective of data feature extraction, without discussing how to improve classification accuracy from the perspective of data feature processing. Is such a plan credible?
