# Supplementary material for: A novel rolling bearing fault detect method based on feature mode decomposition and subtraction-average-based optimizer
Source: PLoS One. 2025 Jun 4;20(6):e0324739. doi: 10.1371/journal.pone.0324739 (PMC12136367; doi:10.1371/journal.pone.0324739)
Supplement: Attachment — Submitted filename: Response to Reviewers.docx [file pone.0324739.s005.docx]

Dear Editor

On behalf of my co-authors, we thank you very much for giving us an opportunity to revise our manuscript, we appreciate editor and reviewers very much for their positive and constructive comments and suggestions on our manuscript entitled “A Novel Rolling Bearing Fault Detect Method Based on Feature Mode Decomposition and Subtrac-tion-Average-Based Optimizer ", (PONE-D-24-49078).

We have studied the Reviewer's comments carefully and have made revision which marked in tracked changes in the revised submission file. We have tried our best to revise our manuscript according to the comments. Attached please find the revised version, which we would like to submit for your kind consideration

We would like to express our great appreciation to you and reviewers for comments on our paper.

Thank you and best regards.

Yours sincerely.

Corresponding author.

Name: Zhang Jingjing

E-mail: zhangjeileen@163.com;

**List of Responses**

Dear Editors and Reviewers:

Thank you for your letter and for the comments concerning our manuscript entitled “A Novel Rolling Bearing Fault Detect Method Based on Feature Mode Decomposition and Subtrac-tion-Average-Based Optimizer ", (PONE-D-24-49078). Those comments are all valuable and very helpful for revising and improving our paper, as well as the important guiding significance to our researches. We have studied comments carefully and have made correction which we hope meet with approval. Revised portion are marked in red in the paper. The main corrections in the paper and the responds to the reviewer's comments are as flowing. The black sections are suggestions and questions from the editors/reviewers, the blue sections are our answers, and the red sections are the revised parts of the paper.

**Responses to the Editor's comments:**

**Responses comments:** Basically, the readability does not meet the criteria, please carefully proof read the contents using professional English writing. (1) Diagram is needed to provide the clear idea of the proposed method (2) while the comparative study is also essential. (3) All typos should be corrected before resubmission along the format correction. (4)

**Response:**

(1) We tried our best to improve the manuscript and made some changes to the manuscript. These changes will not influence the content and framework of the paper. And here we did not list the changes but marked in red in the revised paper. We appreciate for Editors warm work earnestly and hope that the correction will meet with approval.

(2) We appreciate your professional comments on the article. Based on your suggestions, we have added charts and figures to the article to help understand the content and structure of the article. For example, we added a flowchart of the method proposed in the article. (**Fig 1. The flowchart of the proposed method** (Images uploaded separately)) The hardware and parameters used in the experiments, we put them in two tables.

**Modified part:**

**Table 1. The basic parameters of the drive-end bearing**

| **Inside Diameter** | **Outside Diameter** | **Thickness** | **Ball Diameter** | **Pitch Diameter** | **Frequency** |
| --- | --- | --- | --- | --- | --- |
| 0.9843 inches | 2.0472 inches | 0.5906 inches | 0.3126 inches | 1.537 inches | 12000 Hz |

**Table 2. The fault characteristic frequencies** (multiple of running speed in Hz)

| **Inner Ring** | **Outer Ring** |
| --- | --- |
| 5.4152 | 3.5848 |

(3) Thank you for your valuable feedback on the paper. We fully understand the importance of comparative experiments, so we have added an additional set of comparative experiments to the original ones. As shown below, Comparison method 1 is the original comparison method of the article, and Comparison method 2 is the comparison method added according to your suggestion.

**Modified part:**

(From line 263 of the article)

3.2 The Comparison Methods

Two comparison methods are selected to validate the superiority of the proposed method.

Comparison method 1: First, the signal is decomposed using EMD. Next, the kurtosis value of each IMF is calculated. Finally, the IMF with the highest kurtosis value is retained, and its envelope spectrum is obtained.

Comparison method 2: First, two key VMD parameters are set: the penalty factor (alpha = 5000) and the number of modes (k = 5). The signal is then decomposed using VMD. Next, the kurtosis value of each IMF is calculated. Finally, the IMF with the highest kurtosis value is retained, and its envelope spectrum is obtained.

(4) Thank you for highlighting the spelling errors in our manuscript. We realize that such errors can detract from the overall quality of our work. Therefore, we have carefully revised the paper, ensuring that all spelling errors have been corrected. We apologize for any oversight and will strive to avoid such mistakes in future submissions.

**Responds to the Reviewer's comments:**

**Reviewer #1:**

**Response to comment 1:** The authors should cite more references to support the view.

**Response**: We have thoroughly screened and added several key references that directly relate to our research objectives and methodology. We believe this will enrich the discussion and context of our study. We appreciate your attention to detail and hope that the revised manuscript meets your expectations.

**Modified part:**

(From line 57 of the article)

In 2010, Jia-Rong Yeh and Jiann-Shing Shieh proposed complementary ensemble empirical mode decomposition (CEEMD) [15]. CEEMD is an improved algorithm for noise-assisted data analysis. In this method, the residual of added white noise can be extracted from the mixture of data and white noise using pairs of complementary ensemble IMFs with positive and negative white noise. Although this approach yields IMFs with a similar RMS noise level as EEMD, it effectively eliminates residual noise in the IMFs. However, a disadvantage of CEEMD is that redundant IMF components are generated if the amplitude of added white noise and the number of iterations is not appropriately chosen. Therefore, the IMF components need to be recombined or processed.

In 2011, M. E. Torres et al. proposed complete ensemble empirical mode decomposition with adaptive noise (CEEMDAN) [16]. This method has the advantage of requiring less than half the screening iterations of EEMD, and the original signal can be accurately reconstructed by summing the modes. As a result, a smaller ensemble size is required, reducing computational costs. However, this method also has some weaknesses, such as high algorithmic complexity, difficulty in parameter adjustment, sensitivity to noise, and mode mixing issues.

(From line 79 of the article)

In 2017, Li et al. proposed time-varying filtering-based empirical mode decomposition (TVF-EMD) [19]. TVF-EMD is fully adaptive and suitable for analyzing both linear and non-stationary signals. Moreover, it improves frequency separation performance and maintains stability, even at low sampling rates. Additionally, TVF-EMD is robust to noise interference. However, its parameter selection process is not well-defined.

In 2020, Peng et al. proposed broadband mode decomposition (BMD) [20]. BMD is primarily used to analyze complex signals with broadband components, such as square waves and sawtooth waves. However, when applied to variable-speed signals under strong noise interference, BMD exhibits significant deviations. In 2011, to address the limitations of BMD, Geng et al. proposed generalized broadband mode decomposition (GBMD). GBMD combines generalized demodulation with BMD [21].

(From line 99 of the article)

Wang et al. proposed a step-by-step parameter-adaptive FMD method [24]. Experimental results demonstrate that this method is superior in identifying weak periodic fault features. Li et al. proposed an adaptive parameterized FMD method [25]. Experimental results show that this method improves fault diagnosis ac-curacy under strong background noise.

15.	Yeh JR, Shieh JS, Huang NE. Complementary Ensemble Empirical Mode Decomposition: a Novel Noise Enhanced Data Analysis Method. Advances in Adaptive Data Analysis. 2010;2(2):-.

16.	Torres ME, Colominas MA, Schlotthauer G, Flandrin P, editors. A complete ensemble empirical mode decomposition with adaptive noise. International Conference on Acoustics, Speech, and Signal Processing; 2011.

19.	Li H, Li Z, Mo W. A time varying filter approach for empirical mode decomposition. Signal Processing. 2017;138(SEP.):146-58.

20.	Peng Y, Li Z, He K, Liu Y, Li Q, Liu L. Broadband Mode Decomposition and Its Application to the Quality Evaluation of Welding Inverter Power Source Signals. IEEE Transactions on Industrial Electronics. 2020;67:9734-46.

21.	Geng H, Peng Y, Ye L, Guo Y. Generalized broadband mode decomposition method and its application in fault diagnosis of variable speed spherical roller bearing. Measurement. 2023.

24.	Wang X, Li C, Tian H, Xiong X. A step-by-step parameter-adaptive FMD method and its application in fault diagnosis. Measurement Science and Technology. 2024;35(4).

25.	Cong L, Jun Z, Xing W, Tao L. Adaptive feature mode decomposition method for bearing fault diagnosis under strong noise. Proceedings of the Institution of Mechanical Engineers. 2025;239(2):508-19.

**Response to comment 2:** The sections of the article should be numbered, as there is currently a lack of logical organization.

**Response**: Thank you for your valuable suggestions on the article. We believe that numbering the sections of the article is essential and have therefore made the changes as per your suggestions.

**Modified part:**

1 Introduction

2 Method

2.1 Frequency Modulation Decomposition

2.2 Subtraction-Average-Based Optimizer

2.3 Minimum Envelope Entropy

2.4 The Kurtosis

3 The Proposed Method and The Comparison Methods

3.1 The Proposed Method

3.2 The Comparison Methods

4 Data

5 Results and discussion

5.1 Experiment One

5.1.1 Result of Experiment One

5.1.2 Discussion of Experiment One

5.2 Experiment Two

5.2.1 Result of Experiment Two

5.2.2 Discussion of Experiment Two

5.3 Experiment Three

5.3.1 Result of Experiment Three

5.3.2 Discussion of Experiment Three

5.4 Experiment Four

5.4.1 Result of Experiment Four

5.4.2 Discussion of Experiment Four

6 Conclusions

**Response to comment 3:** The article should provide an overall overview of the proposed method, preferably using a combination of images and text to provide a clear introduction.

**Response**: We sincerely appreciate your valuable comments. We also believe that it is important to use a combination of images and text to provide a general overview of the proposed methodology, and have therefore made changes in Chapter 3 of the article.

**Modified part**:

(From line 247 of the article)

3.1 The Proposed Method

This paper proposes a fault diagnosis method. The method employs SABO to optimize FMD parameters, addressing the issue of decomposition performance degradation caused by manual parameter selection in FMD. First, the minimum envelope entropy is used as the fitness function for SABO to determine the optimal combination of filter length (L) and number of modes (n) in FMD. Next, FMD with these parameters de-composes the signal into n IMFs. Subsequently, the IMFs containing fault information are selected based on their maximum kurtosis values. Finally, envelope analysis is applied to identify the fault types in rolling bearings. The steps of the proposed method are as follows, and the flowchart is shown in Fig 1:

**Fig 1. The flowchart of the proposed method.**

Step 1: Load the raw signal and define the parameter ranges for L and n to optimize FMD. Based on reference [22], the range for L is [2, 50], and the range for n is [2, 7].

Step 2: Set the minimum envelope entropy as the fitness function for SABO-optimized FMD.

Step 3: Use SABO to identify the optimal combination of parameters [L, n].

Step 4: Calculate the kurtosis values of the n IMFs and retain the IMF with the highest kurtosis value.

Step 5: Analyze the retained IMFs using envelope spectrum analysis.

**Response to comment 4:** There are many small errors in the article, including spelling and formatting. Additionally, the author should carefully check the formulas and the explanations of symbols within them, such as in Formula (7).

**Response**: Thanks for your careful checks. We are sorry for our carelessness. Based on your comments, the words are carefully checked for spelling and the paper is formatted to ensure that all formatting and spelling errors have been corrected. For formula (7), we apologize for the inadvertent omission of its symbolic interpretation, which we have corrected.

**Modified part**:

(From line 155 of the article)

The correlation coefficient between two modes, $u_{p}$ and $u_{q}$ is defined by equation.

$$CC_{pq}=\frac{\sum_{n=1}^{N} \left(u_{p}\left(n\right)-\overline{u}_{p}\right)\left(u_{q}\left(n\right)-\overline{u}_{q}\right)}{\sqrt{\sum_{n=1}^{N} \left(u_{p}\left(n\right)-\overline{u}_{p}\right)^{2}}\sqrt{\sum_{n=1}^{N} \left(u_{q}\left(n\right)-\overline{u}_{q}\right)^{2}}}$$

Here, $u_{p}$ and $u_{q}$ represent the p-th and q-th decomposition modes, p and q are integers within the interval [1, 2, …, K]. $\overline{u}_{p}$ and $\overline{u}_{q}$ are the mean values of modes $u_{p}$ and $u_{q}$.

**Response to comment 5:** The photo of the experimental setup should be the raw image without any filters, (1) and the hardware and parameter settings used in the experiment should be presented in one or two tables. (2) The reviewer believes that there should be a certain level of progressive relationship from experiments 1 to 4, progressively demonstrating the superiority of the proposed method. (3) The results from the experimental study should also provide the original vibration images. (4) The images should be in vector format, and the text within them is too small and difficult to read. (5) The analysis of the experimental results is not sufficiently in-depth. (6)

**Response**:

（1）Thank you for your advice. We apologize for using the wrong image. Therefore, we have found an unmarked image of the lab equipment at Case Western Reserve University Bearing Data Center. We hope it fits.

**Modified part**:

**Fig 2. The experimental apparatus employed for extracting the experimental data.**

(2) Thank you for your valuable advice. We also think it is very necessary. Therefore, in Chapter 4 of the article, we put the hardware and parameters used in our experiments in two tables.

**Modified part**:

**Table 1. The basic parameters of the drive-end bearing**

| **Inside Diameter** | **Outside Diameter** | **Thickness** | **Ball Diameter** | **Pitch Diameter** | **Frequency** |
| --- | --- | --- | --- | --- | --- |
| 0.9843 inches | 2.0472 inches | 0.5906 inches | 0.3126 inches | 1.537 inches | 12000 Hz |

**Table 2. The fault characteristic frequencies** (multiple of running speed in Hz)

| **Inner Ring** | **Outer Ring** |
| --- | --- |
| 5.4152 | 3.5848 |

(3) Thank you for your kind suggestion. We apologize for any misunderstanding caused to the reviewers by the lack of clarity in the description of the experiments. Actually, the superiority of the proposed method is gradually proved by comparing it with two comparison methods. The decomposition method in the comparison method 1 is EMD without input parameters. The decomposition method in the comparison method 2 is VMD with human input parameters. The decomposition part of the proposed method is FMD after SABO optimization parameters.

For Experiments 1 to 4, they are in fact juxtaposed. We hope to demonstrate the superiority and universality of the method proposed in this paper with the widest possible range of data. As the data used in Experiments 1 to 4, they have different fault types, fault diameters, and motor speeds.

(4) We think that is a good suggestion. Therefore, we supplement the experimental results with the original vibrational images. The following images of a set of experimental results are used as an example.

(5) Thank you for pointing out the image error in the article. We have reformatted the format and resolution of all the images in the article, as well as the format and size of the text in the images. We apologize for any inconvenience caused and will endeavor to avoid such errors in future submissions.

**Modified part**:

(6) Thank you for pointing this out. We also believe that analyzing the results is an important part of the article. Therefore, we have included a separate section for the discussion of each experiment to make the analysis of the results clearer.

**Modified part**:

(From line 309 of the article)

5.1.2 Discussion of Experiment One

The experimental results for the three methods are summarized in Table 4. By comparing the results of the three methods, two conclusions can be drawn. (1) Compared with comparison method 1, the proposed method achieves a lower error rate in fault frequency identification. (2) Although both the comparison method 2 and the proposed method identify the same fault frequencies, the proposed method provides more prominent fault frequency information in the envelope spectrum.

(From line 341 of the article)

5.2.2 Discussion of Experiment Two

The experimental results for the three methods are summarized in Table 6. B By comparing the results of the three methods, two conclusions can be drawn. (1) Compared with the comparison method 1, the pro-posed method achieves a lower error rate in fault frequency identification. (2) Although the comparison method 2 identifies more frequency components, the proposed method provides more prominent fault frequency information in the envelope spectrum.

(From line 374 of the article)

5.3.2 Discussion of Experiment Three

The experimental results for the three methods are summarized in Table 8. By comparing the results of the three methods, two conclusions can be drawn. (1) Compared with the comparison method 1, the pro-posed method achieves a lower error rate in fault frequency identification. (2) Compared with the comparison method 2, the proposed method extracts more effective fault-related information.

(From line 406 of the article)

5.4.2 Discussion of Experiment Four

The experimental results for the three methods are summarized in Table 10. By comparing the results of the three methods, two conclusions can be drawn. (1) Compared with the comparison method 1, the pro-posed method obtains lower error rate of the information. (2) Although both the proposed method and the comparison method 2 identify the same fault frequencies, the proposed method provides more prominent fault-related information.

**Response to comment 6:** The conclusion should be supported by relevant data, and it should also introduce the advantages and innovative aspects of the proposed methods.

**Response**: Thank you for your insightful suggestion. We apologize for the immaturity of the textual descriptions of the experimental and conclusion sections. In fact, the results of the 12 sets of experiments in the paper support the conclusions reached in the paper. (A detailed discussion of the results and experiments is given in Chapter 5 of the paper.) At the same time, without affecting the content of the article, we have rewritten the conclusion section, and hope that the revision will meet your expectations.

**Modified part**:

6 Conclusions

This paper proposes a fault diagnosis method based on SABO and FMD. The method first employs SABO to optimize FMD parameters, then uses kurtosis values to screen IMFs containing fault information, and finally retains the envelope spectrum of the selected IMF to extract fault frequency information.

The main contributions of this paper are as follows:

(1) The proposed method effectively addresses the issue of manual parameter selection in FMD de-composition, improving decomposition efficiency.

(2) The proposed method uses kurtosis values to effectively screen IMFs containing fault information, reducing computational costs and improving fault diagnosis efficiency.

(3) Compared with other similar algorithms, the proposed method offers a lower fault identification error rate and more distinct fault characteristics.

(4) The proposed method accurately and efficiently identifies fault information for different fault types, fault diameters, and motor speeds, demonstrating its potential for widespread application in fault diagnosis.

The method provides a novel approach to fault extraction, improving fault diagnosis accuracy and reducing computational costs. Future research will focus on developing more effective fault extraction and diagnosis methods for rolling bearings, as well as accurate classification techniques for extracted fault information.

**Response to comment 7:** The format of the references is confused.

**Response**: Thank you for pointing out the problem with the references in the text. We are aware that such errors may affect the overall quality of the article. Therefore, we have re-imported the references using Endnote software to meet the formatting requirements. Once again, we apologize for our oversight. Here are the references for the article, hopefully they meet the requirements

**Modified part**:

**References**

1.	Liu ZL, Xingqiu. Rolling bearing fault diagnosis using variational autoencoding generative adversarial networks with deep regret analysis. Measurement. 2021;168(1).

2.	Cui L, Sun Y, Wang X, Wanga H. Spectrum-Based, Full-Band Preprocessing, and Two-Dimensional Separation of Bearing and Gear Compound Faults Diagnosis. IEEE Transactions on Instrumentation and Measurement. 2021;PP(99):1-.

3.	Chang Z, Jia Q, Yuan X, Chen Y. Main failure mode of oil-air lubricated rolling bearing installed in high speed machining. TRIBOLOGY INTERNATIONAL. 2017;112:68-74.

4.	Jardine AKS, Lin D, Banjevic D. A review on machinery diagnostics and prognostics implementing condition-based maintenance. Mechanical Systems and Signal Processing. 2006;20.

5.	Randall RB. Vibration-based Condition Monitoring: Industrial, Aerospace and Automotive Applications: Vibration-based condition monitoring : industrial, aerospace and automotive applications; 2011.

6.	D FCAB, B MCA, C BT, A WX, A BC, B XS. A novel optimized multi-kernel relevance vector machine with selected sensitive features and its application in early fault diagnosis for rolling bearings. Measurement. 156.

7.	Tian Q, Hou C, Li S. Adaptive resonant demodulation for early fault diagnosis of ball bearing. IEEE. 2013.

8.	Wang X, Zi Y, He Z. Multiwavelet denoising with improved neighboring coefficients for application on rolling bearing fault diagnosis. Mechanical Systems & Signal Processing. 2011;25(1):285-304.

9.	Leite VCMN, Borges dS, J. G, Veloso GFC, Borges dS, L. E, Lambert-Torres G, Bonaldi EL, et al. Detection of Localized Bearing Faults in Induction Machines by Spectral Kurtosis and Envelope Analysis of Stator Current. Industrial Electronics IEEE Transactions on. 2015;62(3):1855-65.

10.	Zheng K, Li T, Su Z, Zhang B. Sparse Elitist Group Lasso Denoising in Frequency Domain for Bearing Fault Diagnosis. IEEE Transactions on Industrial Informatics. 2021;17(7):4681-91.

11.	Miao Y, Wang J, Zhang B, Li H. Practical framework of Gini index in the application of machinery fault feature extraction. Mechanical Systems and Signal Processing. 2022;165:108333.

12.	Ne. H, Sr. L, Mlc. W, Hh. S, Qn. Z, Nc. Y, et al. The empirical mode decomposition and the Hilbert spectrum for nonlinear and non-stationary time series analysis. Proceedings of the Royal Society Mathematical, physical and engineering sciences. 1998;(1971):454.

13.	Smith, Jonathan S. The local mean decomposition and its application to EEG perception data. Journal of The Royal Society Interface. 2005;2(5):443-54.

14.	Wu Z, Huang NE. ENSEMBLE EMPIRICAL MODE DECOMPOSITION: A NOISE-ASSISTED DATA ANALYSIS METHOD. ADVANCES IN DATA SCIENCE AND ADAPTIVE ANALYSIS. 2009;1(01):1-41. doi: Doi:10.1142/s1793536909000047.

15.	Yeh JR, Shieh JS, Huang NE. Complementary Ensemble Empirical Mode Decomposition: a Novel Noise Enhanced Data Analysis Method. Advances in Adaptive Data Analysis. 2010;2(2):-.

16.	Torres ME, Colominas MA, Schlotthauer G, Flandrin P, editors. A complete ensemble empirical mode decomposition with adaptive noise. International Conference on Acoustics, Speech, and Signal Processing; 2011.

17.	Gilles J. Empirical Wavelet Transform. IEEE Trans Signal Processing. 2013;61(16):3999-4010.

18.	Dragomiretskiy K, Zosso D. Variational Mode Decomposition. IEEE Transactions on Signal Processing. 2014;62:531-44.

19.	Li H, Li Z, Mo W. A time varying filter approach for empirical mode decomposition. Signal Processing. 2017;138(SEP.):146-58.

20.	Peng Y, Li Z, He K, Liu Y, Li Q, Liu L. Broadband Mode Decomposition and Its Application to the Quality Evaluation of Welding Inverter Power Source Signals. IEEE Transactions on Industrial Electronics. 2020;67:9734-46.

21.	Geng H, Peng Y, Ye L, Guo Y. Generalized broadband mode decomposition method and its application in fault diagnosis of variable speed spherical roller bearing. Measurement. 2023.

22.	Miao Y, Zhang B, Li C, Lin J, Zhang D. Feature Mode Decomposition: New Decomposition Theory for Rotating Machinery Fault Diagnosis. IEEE Transactions on Industrial Electronics. 2023;70:1949-60.

23.	Lei H, Wang JY, Zheng JW, Hou XD, Ding JM. Early fault diagnosis of rolling bearings based on poa optimised fmd parameters. Failure Analysis and Prevention. 2024;19(4):264-72. doi: 10.3969/j.issn.1673-6214.2024.04.006.

24.	Wang X, Li C, Tian H, Xiong X. A step-by-step parameter-adaptive FMD method and its application in fault diagnosis. Measurement Science and Technology. 2024;35(4).

25.	Cong L, Jun Z, Xing W, Tao L. Adaptive feature mode decomposition method for bearing fault diagnosis under strong noise. Proceedings of the Institution of Mechanical Engineers. 2025;239(2):508-19.

26.	Mcdonald GL, Zhao Q, Zuo MJ. Maximum correlated Kurtosis deconvolution and application on gear tooth chip fault detection. Mechanical Systems and Signal Processing. 2012;33(Complete):237-55.

27.	Zhao M, Lin J, Miao Y, Xu X. Detection and recovery of fault impulses via improved harmonic product spectrum and its application in defect size estimation of train bearings. Measurement. 2016;91:421-39.

28.	Trojovsk P, Dehghani M. Subtraction-Average-Based Optimizer: A New Swarm-Inspired Metaheuristic Algorithm for Solving Optimization Problems. Biomimetics (2313-7673). 2023;8(2).

29.	Jiang Y, Tang B, Qin Y, Liu W. Feature extraction method of wind turbine based on adaptive Morlet wavelet and SVD. Renewable Energy. 2011;36(8):2146-53. doi: <https://doi.org/10.1016/j.renene.2011.01.009>.

30.	Wang Z, He G, Du W, Zhou J, Han X, Wang J, et al. Application of Parameter Optimized Variational Mode Decomposition Method in Fault Diagnosis of Gearbox. IEEE Access. 2019;7:44871-82. doi: 10.1109/ACCESS.2019.2909300.

**Reviewer #2:**

**Response to comment a:** The paper's format is not standardized enough, for example, there are deformations in the symbols and letters, the font in the figures is too small, and there are multiple blanks in the paper.

**Response**: Thank you for pointing out the errors in the manuscript. We realize that they may have affected the overall quality of the article. Therefore, we have double-checked the formatting of the article, the formulas in the article, and so on. At the same time, we have updated all the images in the article, including the resolution and size of the images and the formatting of the text in the images. We apologize for these problems and hope that our changes meet your expectations. Picture is as follows.

**Modified part**:

**Response to comment b:** There is a lack of an overall scheme diagram, which reduces the readers' understanding of the methodological process of the paper.

**Response**: Thank you for your kind suggestion. We also believe that the addition of a scheme diagram will help the reader to understand the method proposed in this paper. Therefore, we add the flowchart of the proposed method in Chapter III. We hope this improvement meets your expectations.

**Modified part**:

(From line 247 of the article)

3.1 The Proposed Method

This paper proposes a fault diagnosis method. The method employs SABO to optimize FMD parameters, addressing the issue of decomposition performance degradation caused by manual parameter selection in FMD. First, the minimum envelope entropy is used as the fitness function for SABO to determine the optimal combination of filter length (L) and number of modes (n) in FMD. Next, FMD with these parameters de-composes the signal into n IMFs. Subsequently, the IMFs containing fault information are selected based on their maximum kurtosis values. Finally, envelope analysis is applied to identify the fault types in rolling bearings. The steps of the proposed method are as follows, and the flowchart is shown in Fig 1:

Step 1: Load the raw signal and define the parameter ranges for L and n to optimize FMD. Based on reference [22], the range for L is [2, 50], and the range for n is [2, 7].

Step 2: Set the minimum envelope entropy as the fitness function for SABO-optimized FMD.

Step 3: Use SABO to identify the optimal combination of parameters [L, n].

Step 4: Calculate the kurtosis values of the n IMFs and retain the IMF with the highest kurtosis value.

Step 5: Analyze the retained IMFs using envelope spectrum analysis.

**Fig 1. The flowchart of the proposed method.**

**Response to comment c:** The paper's methodology focuses on achieving accurate classification of rolling bearing faults from the perspective of data feature extraction, without discussing how to improve classification accuracy from the perspective of data feature processing. Is such a plan credible?

**Response**: Thank you for your insightful questions. Allow us to explain this.

The main emphasis of this paper is on feature extraction, as the quality of the extracted features is the basis for any fault diagnostic system to be able to successfully classify faults for accurate discrimination. We use the method proposed in this paper to obtain the kurtosis value of IMF containing fault information, which will become the key to accurate classification. Without effective feature extraction, even advanced feature processing techniques may not be able to significantly improve the classification accuracy.

With respect to scheme credibility, we believe that the experimental results in this paper support the credibility of our approach. This paper shows the experimental results of 12 sets of experiments. The results are presented for 4 different fault types, fault diameters, and motor speeds. Each set of experimental results contains the results of experiments using the method proposed in this paper and the results of experiments using two comparative methods. By observing and analyzing the experimental results, we find that the method proposed in this paper outperforms the two comparative methods in any set of experiments. Meanwhile, through our previous experimental results as well as extensive reading of the literature, we found that the higher the accuracy of the results obtained by the adopted fault feature extraction method, the higher the accuracy of the subsequent classification of fault information using the feature values obtained by the method.

We recognize your point of view and it is a good suggestion for the future. We plan to extract the eigenvalues using the methodology proposed in this paper and subsequently conduct fault classification experiments using the obtained eigenvalues.

In conclusion, while our paper primarily focuses on feature extraction, the proposed methodology is credible and effective, as evidenced by the experimental results. We appreciate the reviewer's feedback and will consider incorporating feature processing techniques in future research to further enhance the performance of our fault diagnosis system. Thank you for your valuable input!

**Other changes**

(1) In order to help readers read more clearly, we have made some adjustments to the structure of the article without changing its content. For example, a separate chapter on the methodology proposed in this paper and the two comparative methods; and separation of the results and discussion of each experiment in chapter 5.

(2) We have updated all the formulas in the article.

(3) In Chapter 5, we have added four tables that illustrate the experimental data to help the reader visualize the experimental data more intuitively.

We tried our best to improve the manuscript and made some changes in the manuscript. These changes will not influence the content of the paper. And here we did not list the changes but marked in red in revised paper. We appreciate for Editors/Reviewers’warm work earnestly, and hope that the correction will meet with approval. Once again, thank you very much for your comments and suggestions.

Thank you and best regards.

Yours sincerely.

Zhang Jingjing

14 Feb 2025
